# Trends in the diagnostic delay and pathway for amyotrophic lateral sclerosis patients across different countries

**DOI:** 10.3389/fneur.2022.1064619

**Published:** 2023-01-17

**Authors:** Catarina Falcão de Campos, Marta Gromicho, Hilmi Uysal, Julian Grosskreutz, Magdalena Kuzma-Kozakiewicz, Miguel Oliveira Santos, Susana Pinto, Susanne Petri, Michael Swash, Mamede de Carvalho

**Affiliations:** ^1^Instituto de Fisiologia, Instituto de Medicina Molecular, Centro de Estudos Egas Moniz, Faculdade de Medicina, Universidade de Lisboa, Lisbon, Portugal; ^2^Department of Neurosciences and Mental Health, Centro Hospitalar Universitário de Lisboa-Norte, Lisbon, Portugal; ^3^Department of Neurology and Clinical Neurophysiology, Akdeniz University Faculty of Medicine, Antalya, Turkey; ^4^Hans Berger Department of Neurology, Jena University Hospital, Jena, Germany; ^5^Neurodegenerative Disease Research Group, Medical University of Warsaw, Warsaw, Poland; ^6^Department of Neurology, Hannover Medical School, Hannover, Germany; ^7^Department of Neurology and Neuroscience, Barts and the London School of Medicine, Queen Mary University of London, London, United Kingdom

**Keywords:** amyotrophic lateral sclerosis, motor neuron disease, diagnostic delay, diagnostic pathway, time to diagnosis

## Abstract

**Background:**

Amyotrophic lateral sclerosis (ALS) is a rapidly progressive neurodegenerative disease with a median survival of 2–5 years. An early diagnosis is essential for providing ALS patients the finest management possible. Studies from different countries report a similar median diagnostic delay of around 12 months, which is still far from desirable. We analyzed the diagnostic pathway in different countries in order to identify the major challenges.

**Methods:**

We studied a cohort of 1,405 ALS patients from five different centers, in four different countries (Turkey, Germany, Poland, and Portugal), which collaborated in a common database. Demographic, disease and sociocultural factors were collected. Time from first symptom onset to first medical evaluation and to diagnosis, the specialist assessment and investigations requested were analyzed. Factors contributing to diagnostic delay were evaluated by multivariate linear regression.

**Results:**

The median diagnostic delay from first symptom onset was 11 months and was similar between centers. Major differences were seen in the time from symptom onset to first medical evaluation. An earlier first medical evaluation was associated with a longer time to diagnosis, highlighting that ALS diagnosis is not straightforward in the early stages of the disease. The odds for ALS diagnosis were superior when evaluated by a neurologist and increased over time. Electromyography was decisive in establishing the diagnosis.

**Conclusions:**

We suggest that a specific diagnostic test for ALS—a specific biomarker—will be needed to achieve early diagnosis. Early referral to a neurologist and to electromyography is important for early ALS diagnosis.

## Introduction

Amyotrophic lateral sclerosis (ALS) is a neurodegenerative disease characterized by loss of motor neurons, leading to progressive weakness and wasting. ALS often has a focal onset before spreading to other body regions, culminating in death due to respiratory failure (1). Diagnosis in the early stages of the disease remains challenging. This is related to clinical heterogeneity and, especially, lack of a specific diagnostic biomarker (2). The clinician is therefore inclined to search for other treatable entities before establishing this devastating diagnosis. These factors all contribute to the mean reported diagnostic delay of around 12 months from symptom onset, which is far from acceptable considering its median survival of 2–5 years from disease onset (1, 3–6). Although there is no effective treatment, a prompt diagnosis is critical for several reasons. First, allows for an early referral to centers offering multidisciplinary management, which has been shown to increase survival and improve quality of life for ALS patients (7, 8). Second, a delayed diagnosis enhances the psychological distress associated with uncertain diagnosis leading to added social and financial concerns. Third, a late diagnosis may prevent a patient's inclusion in a clinical trial (9). Fourth, diagnosis uncertainty may lead to unnecessary interventions, such as surgery (9, 10), which can hasten disease progression (11).

Efficient access to healthcare, and primarily to a neurology clinic specialized in neuromuscular disorders, seemingly would be important in shortening the time to diagnosis (9, 12, 13). However, although national referral systems and healthcare institutions vary between different countries, a similar time to diagnosis has been reported from studies conducted in distinct centers worldwide, and has remained unchanged during 20 years (3–6). Nevertheless, previous studies were developed independently using divergent methodology or registries. Here, we analyze the diagnostic delay and pathway of ALS patients from five ALS centers in four countries, which collaborated on the *OnWebDUALS* project and used a standard database (14).

## Materials and methods

### Study population

We studied adult ALS patients diagnosed and registered on the project's database, between January 2015 and July 2021, from five ALS centers in four different countries (Antalya, Turkey; Hannover and Jena, Germany; Warsaw, Poland; and Lisbon, Portugal). Patients with definite, probable, probable laboratory-supported and possible ALS, according to the revised El-Escorial criteria (rECC) were included (15). Patients with progressive muscular atrophy (PMA) and progressive bulbar palsy (PBP) were also included in the study, as both are accepted phenotypes of ALS (16). However, patients with monomelic motor neuron disease, Kennedy disease and primary lateral sclerosis were not included, given the different pattern of progression of these disorders. Missing data concerning the dates of symptom onset and diagnosis were also exclusion criteria. Additionally, patients who did not consent to participate in the study or were unable to provide reliable information regarding their diagnostic pathway, even with the caregivers' contribution, were excluded. Other disorders, associated by chance with the ALS syndrome, did not lead to patient exclusion.

### Data collection

Demographic and clinical data were collected at the first visit to the ALS clinic by strictly applying a standardized questionnaire developed in the project, as published elsewhere (14). Place of living (rural vs. urban areas) and main occupations before disease onset, classified according to the International Standard Classification of Occupations (ISCO), were also obtained. The average monthly income was estimated, using the ILOSTAT database of the ILO (International Labor Organization). The income variable was then transformed to a binominal variable, employing the calculated median as cut-off. Gross domestic product (GDP) per capita in the different reference centers' regions was collected from the Organization for Economic Co-operation and Development (OECD) database (OECD.stat). Diagnostic delay was determined from symptom onset to diagnosis. To describe the diagnostic pathway of ALS patients, data regarding time from first symptom to first medical evaluation, number of medical evaluations, medical specialists involved (neurologist vs. non-neurologist) until diagnosis, and investigations requested (including CT or MR imaging and neurophysiological studies) were analyzed. To evaluate the effect of rate of functional decline on diagnostic delay, the ALSFRS-R (ALS functional rating score) decline rate was calculated (48—ALSFRS-R at study entry/number of months since first symptoms). The diagnostic pathway was further explored in the five different participating centers.

The project was approved by the locals Ethical Committees. All patients gave written informed consent before inclusion in the study.

### Statistical analysis

Data analysis was performed with STATA13 software. For descriptive analysis, means and medians, with standard deviations and interquartile ranges respectively, were calculated for continuous variables, and percentages for categorical variables. Predictors of diagnostic delay were identified using uni- and multivariate linear regression models. Predictors strongly associated with the outcome in univariate models were included in the final model. The following predictors were evaluated: age at disease onset, gender, predominant lower motor neuron or/and upper motor neuron at disease onset, bulbar vs. spinal-onset, ALSFRS rate of decay, presence of cognitive symptoms at onset, family history of ALS or frontotemporal dementia (FTD), center where the patient was evaluated, place of living (rural vs. urban area), monthly income, GDP per capita in the different reference centers' regions and co-morbidities such as previous stroke, diabetes or spinal surgery. The one-way ANOVA test was used to compare continuous variables and the Chi-squared test to compare categorical data between patients from different ALS centers. A *p*-value < 0.05 was considered statistically significant.

## Results

### Patients

From the initial 1,590 patients registered in the database, 185 were excluded due to missing diagnostic and symptom onset dates. A final cohort of 1,405 ALS patients was studied with a mean age at disease onset of 59.7 ± 13.7 years; 57% were male. Most patients (638; 45%) were registered in the Lisbon ALS center, followed by Warsaw (276; 20%), Hannover (205; 14%), Antalya (179; 13%) and Jena (107; 8%) ALS centers. The majority of patients were classified as definite and probable/probable lab-supported ALS according to the rEEC (15) (27 and 33%, respectively); 15% of patients were diagnosed with PMA and 2% with PBP. One quarter (24%) of patients had a bulbar onset. Baseline characteristics of ALS patients from each center are shown in Table 1.

**Table 1:** Baseline characteristics and diagnostic features of ALS patients from each European center.

	**Antalya** **(*n* = 179; 13%)**	**Hannover** **(*n* = 205; 14%)**	**Jena** **(*n* = 107; 8%)**	**Lisbon** **(*n* = 638; 45%)**	**Warsaw** **(*n* = 276; 20%)**	***p*-value**
Age at disease onset (years)	55.0 ± 13.5	60.8 ± 11.3	62.5 ± 12.2	62.3 ± 13.5	55.2 ± 14.8	**< 0.001** ^**a**^
Gender (male)	113 (63%)	129 (63%)	60 (56%)	367 (57%)	139 (50%)	**0.031** ^**a**^
**Predominant UMN vs. LMN at onset in ALS patients**						
Predominant UMN	17%	10%	27%	31%	10%	**< 0.001** ^**b**^
Predominant LMN	54%	84%	71%	67%	86%	
No predominant UMN/LMN	29%	6%	2%	2%	4%	
Bulbar-onset	25%	26%	40%	22%	28%	**0.001** ^**b**^
ALSFRS-R rate of decline (per month)	0.9 ± 1.3	0.7 ± 0.8	0.7 ± 0.8	0.8 ± 0.8	0.9 ± 2.6	0.510^b^
Clinical cognitive dysfunction at onset	16%	18%	13%	7%	7%	**< 0.001** ^a^
Positive ALS/FTD family history	9%	11%	7%	8%	9%	0.636^a^
**Place of living**						
Rural area	36%	54%	0	13%	31%	**< 0.001** ^a^
Urban area	64%	46%	100%	87%	69%	
**Monthly average income (euros)**						
< 1,064	69%	1%	0%	69%	56%	**< 0.001** ^a^
≥1,064	31%	99%	100%	31%	44%	
Mean and median diagnostic delay (months, 1^st^-3^rd^ IQR)^*^	19.1 ± 26.1	15.4 ± 16.7	15.6 ± 21.3	16.7 ± 21.0	21.0 ± 31.3	**0.042** ^b^
	11 (6–24)	11 (6–18)	10 (7–17)	10 (6–18)	12 (6–24)	
Mean and median time gap between symptoms onset and first medical evaluation (months, 1^st^-3^rd^ IQR)^*^	8.1 ± 15.8	5.9 ± 12.6	6.3 ± 9.7	5.2 ± 7.9	4.7 ± 6.7	**0.004** ^b^
	3 (1–9)	2 (1–6)	4 (1–7)	3 (1–6)	3 (1–6)	
Mean and median time gap between first medical evaluation and diagnosis (months, 1^st^-3^rd^ IQR)^*^	11.3 ± 20.9	9.7 ± 10.0	8.9 ± 17.8	11.0 ± 19.1	16.3 ± 29.5	**0.002** ^b^
	6 (1–12)	7 (3–13)	5 (1–10)	5 (2–12)	8 (3–19)	

ALS, amyotrophic lateral sclerosis; ALSFRS-R, ALS functional rating scale revised; FTD, frontotemporal dementia; LMN, lower motor neuron; UMN, upper motor neuron.

^a^Chi-squared test, ^b^One-way ANOVA test.

^*^The mean diagnostic delay, mean time gap from first symptoms onset and mean first medical evaluation and time gap from first medical evaluation and diagnosis difference between each centers are presented in the Supplementary Tables.

The bold values indicate the statistically significant (*p*-value < 0.05).

### Diagnostic delay and predictors

In this cohort the median diagnostic delay from first symptom onset was 11 months (1st−3rd IQ = 6–20) and the average diagnostic delay was 17.6 ± 23.6 months. Diagnostic delay was slightly higher in Antalya and Warsaw centers but no major significant difference was seen between centers (Table 1). Diagnostic delay was also evaluated in patients classified as possible ALS according to rEEC. Likewise, the median diagnostic delay was 11 months (1st−3rd IQ = 6–24) and no difference was found between centers 12 months (1st−3rd IQ = 6–24) in Antalya, 12 months (1st−3rd IQ = 5–20) in Hannover, 12 months (1st−3rd IQ = 9–67) in Jena, 8.5 months (1st−3rd IQ = 5–18) in Lisbon and 11 months (1st−3rd IQ = 6–26) in Warsaw; *p* = 0.23).

In the univariate analyses the following predictors were significant and further included in the multivariate analysis: age at disease onset (coef −0.35, *p* < 0.001) bulbar onset (coef −6.09, *p* < 0.001), ALSFRS rate of decay (−3.59, *p* < 0.001) and center where the patients were evaluated compared to the Warsaw center (Antalya coef −1.94; *p* = 0.39; Hannover coef −5.57, *p* = 0.01; Jena coef −5.43, *p* = 0.04; Lisbon coef −4.31, *p* = 0.01). The remaining evaluated variables were not significant predictors of diagnostic delay.

In the multivariate linear regression analysis, younger patients were diagnosed significantly later (coef −5.49, *p* < 0.001). Patients with bulbar-onset and faster disease progression (higher ALSFRS-R rate of decline) were linked to a shorter diagnostic delay (coef. −3.78, *p* < 0.001; coef. −6.87, *p* < 0.01, respectively). Patients seen in Antalya, Hannover, Jena and Lisbon centers were not diagnosed significantly later than patients seen in the Warsaw center (coef. −2.32, *p* = 0.29; coef. −4.22, *p* = 0.05; coef. −3.06, *p* = 0.24; coef −2.64, *p* = 0.12 respectively). Gender, predominant lower motor neuron or/and upper motor neuron at disease onset, cognitive symptoms at onset, family history of ALS and FTD, place of living and monthly income were not predictors of diagnostic delay. The mean diagnostic delay was also not affected by known comorbidities such as stroke, diabetes or previous spinal surgery. Predictors of diagnostic delay for each center were also explored (Table 2). Faster disease progression was the most important factor associated with a shorter time until diagnosis.

**Table 2:** Multivariate linear regression analysis assessing predictors of diagnostic delay in ALS patients from each European center.

	**Antalya**		**Hannover**		**Jena**		**Lisbon**		**Warsaw**	
	**Coef**	***p*-value**	**Coef**	***p*-value**	**Coef**	***p*-value**	**Coef**	***p*-value**	**Coef**	***p*-value**
Age at disease onset	−0.17	0.19	0.04	0.69	−0.03	0.85	–**0.16**	**0.01**	–**0.55**	**< 0.001**
Sex	3.80	0.30	−1.96	0.39	1.83	0.67	−1.10	0.49	4.91	0.22
Bulbar	−4.10	0.33	–**5.24**	**0.04**	−3.87	0.41	–**5.73**	**< 0.001**	−5.90	0.24
ALSFRSR rate of decay	–**4.46**	**< 0.001**	–**7.06**	**< 0.001**	–**5.51**	**0.04**	–**7.98**	**< 0.001**	−1.18	0.13

ALS, amyotrophic lateral sclerosis; ALSFRS-R, ALS functional rating scale revised. The bold values indicate the statistically significant (p-value < 0.05).

### Diagnostic pathway

**Figure 1:**
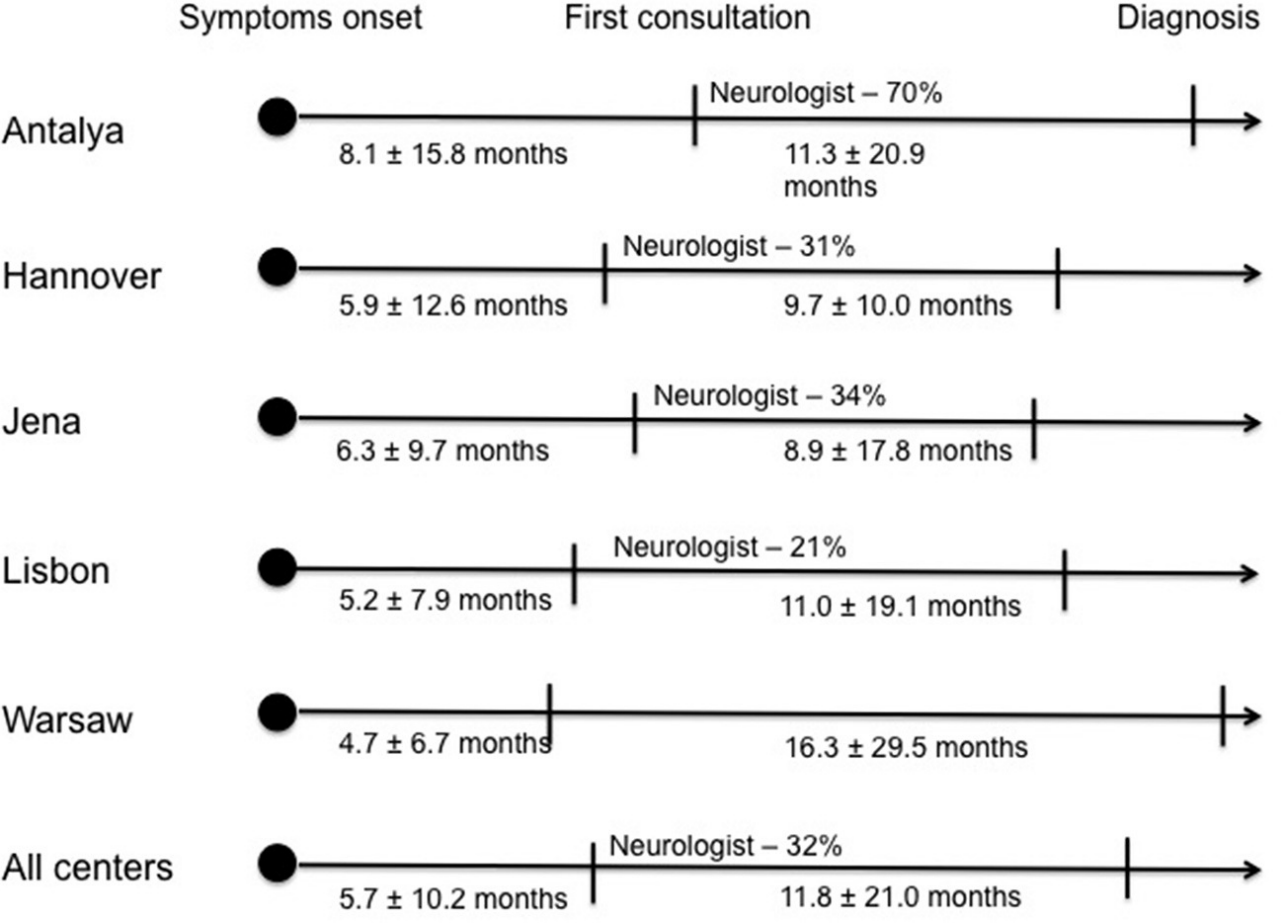
Diagnostic pathway of ALS patients in each center: Time from first symptom onset to first medical evaluation and time from first medical evaluation to final diagnosis; proportion of Neurologists who observed the patient at first medical evaluation.

The median time between symptom onset and first medical evaluation in our cohort was 3 months (1st−3rd IQ = 1–6) and the mean was 5.7 ± 10.2 months. The time from first medical consultation to final diagnosis represents the only modifiable variable; the median time was 6 months (1st−3rd IQ = 2–13) and the mean time was 11.8 ± 21.0 months. Patients from Antalya took longer time to consult a first physician after symptom onset (average time of 8.1 ± 15.8 months), compared to other centers. However, the time to diagnosis after the first medical evaluation was similar. In contrast, patients from Warsaw center were able to arrange a first medical evaluation earlier (average time of 4.7 ± 6.7 months). Nonetheless, the time until diagnosis from first consultation was longer in Warsaw compared to the other centers (average time of 16.3 ± 29.5 months) (Figure 1, Table 1). Considering the first specialist involved, the majority of patients seen at the Antalya center first consulted a neurologist (70%) but fewer of the patients from the remaining centers were assessed first by a neurologist (31% in Hannover, 34% in Jena, 21% in Lisbon and 35% in Warsaw) (Figure 1). The non-neurologists first assessing the patients were mainly general practitioners, orthopedists, neurosurgeons and otolaryngologists, the latter primarily in bulbar-onset patients.

The overall chance of ALS diagnosis was far superior in patients who were observed by a neurologist vs. a non-neurologist physician (95 vs. 5%). However, the majority of patients who first consulted a neurologist were not immediately diagnosed (24% neurologists diagnosed ALS at first evaluation). Additionally, electromyography (EMG) was only performed in 43% of the patients who were first observed by a neurologist. Nonetheless, the likelihood of diagnosis further increased in the following consultations. Patients from Warsaw consulted a median of four different specialists until diagnosis, while patients from Antalya consulted two and the remaining patients from the other centers three specialists until diagnosis. The main investigation requested by the specialist who made the diagnosis was an EMG, performed in 90% of the patients. Imaging was also often requested, namely brain MRI (37%) and cervical MRI (33%).

## Discussion

In our multinational cohort the median diagnostic delay was 11 months as has been previously reported (3–6, 12). The shortest mean time from first symptoms to clinical diagnosis was 6.8 ± 6.1 months reported from an epidemiological study in Southern Germany (17).

Considering the diagnostic delay for each center no major differences were seen. However, the diagnostic pathway differed between centers, with patients from Antalya center having late access to a first medical evaluation and patients from Warsaw an early access. Furthermore, the first specialist assessing the patients also differed, with the majority of patients from Antalya consulting a neurologist but patients in the remaining centers first consulting a non-neurologist specialist. These findings may be justified by each country's healthcare organization with different patterns of access to medical assessment and referral to a specialist, namely a neurologist.

Examining the predictors of diagnostic delay, faster disease progression was irrefutably associated to a shorter diagnostic delay, as previously described (12, 13, 18–21). Bulbar-onset was also independently associated to a shorter diagnostic delay but only in the Hannover and Lisbon centers. In previous studies, site of onset has been reported as a significant predictor of diagnostic delay with bulbar-onset patients being diagnosed more promptly (13, 18–20). It is recognized that bulbar-onset ALS patients present with more rapid decline and shorter survival since disease onset, comparing to spinal-onset ALS patients. Thus, in the multivariate linear regression analysis, when adjusting for disease progression as a potential confounder, site of onset was no longer a significant predictor of diagnostic delay in some centers.

Younger age at disease onset was associated with an increased diagnostic delay in the Lisbon and Warsaw centers, as reported elsewhere (20). This is probably associated with the greater challenge for diagnosis of a neurodegenerative disease in an age group rarely affected, the consequent need to perform more investigations, and a greater wish to offer treatments (like as immunoglobulins) before a final diagnosis is made. Remarkably, at the Warsaw center, faster disease progression was not significantly associated with a shorter diagnostic delay. In the Warsaw center, since patients rapidly obtained a medical assessment (4.7 ± 6.7 months), they were observed in the earlier stages of the disease presumably with minor suggestive signs and symptoms, making the diagnosis more challenging. Therefore, we anticipate that the risk of misdiagnosis was greater, leading to more investigations and interventions and subsequent increased time to diagnosis, as shown in Table 1. Regardless of the time to the first medical evaluation, the time to diagnosis was similar in patients between centers.

These findings emphasize that ALS diagnosis is not straightforward in the early stages of the disease. El Escorial criteria, as diagnostic criteria for ALS, have been used in clinical practice for almost three decades, with the revision in 2000 (15) and the Awaji modification in 2008 (22). Despite these revisions, these criteria still lack sensitivity (23, 24), and suffer from complexity (25). To overcome these limitations, a new set of simpler criteria has been proposed—Gold Coast Criteria (26). These new criteria have higher sensitivity with similar specificity comparing to rECC, that was maintained in the different clinical subgroups defined by site of onset and by disease duration (27, 28). In addition to the development of new criteria, early referral to a neurologist is also of paramount importance in improving ALS diagnosis (4, 13, 21). Surprisingly, only a minority of patients who first consulted a neurologist were immediately diagnosed. Possibly, the neurologists tended to search for other treatable or benign conditions and deferred establishing this devastating diagnosis. Also, only 43% of the neurologists requested an EMG study, which may explain the lower than expected ratio of diagnosis. As demonstrated in our study, neurophysiological evaluation was a decisive investigation accounting for 90% of the ALS diagnoses.

Our study has some limitations. First, our results may be biased since almost half of our cohort came from the Lisbon center. However, we also analyzed the diagnostic delay and pathway for each center with similar findings. Second, although patients who were unreliable in providing information regarding their diagnostic pathway were excluded, the risk of recall bias cannot be neglected.

Although ALS patients from each center had different diagnostic pathways, the diagnostic delay was not outstandingly different, which means that it might be independent of each country's national referral systems and healthcare organizations. The median diagnostic delay remains disappointing. We believe that the Gold Coast criteria may assist in reducing diagnostic delay by identifying ALS patients in the early stages of disease due to its higher sensitivity and simplicity comparing to rECC (27, 28). Early referral to a neurologist and, especially, early referral for EMG studies are critical for a prompt diagnosis.

## Data availability statement

All analyses and anonymized data will be shared by request from any qualified investigator and after consideration of the scientific project.

## Ethics statement

The studies involving human participants were reviewed and project was approved by the Local Ethical Committees. All patients gave written informed consent before inclusion in the study. The patients/participants provided their written informed consent to participate in this study.

## Author contributions

CF: data analysis and manuscript writing. MG: data collection and manuscript writing. HU, JG, MK-K, MO, SPi, SPe, MC, and MS: data collection and manuscript review. MC: idea concept. All authors contributed to the article and approved the submitted version.
