# Supplementary material for: Trends in the diagnostic delay and pathway for amyotrophic lateral sclerosis patients across different countries
Source: Front Neurol. 2023 Jan 17;13:1064619. doi: 10.3389/fneur.2022.1064619 (PMC9886675; doi:10.3389/fneur.2022.1064619)
Supplement: Supplementary file 1 [file Table_1.DOCX]

Supplementary tables

**Table 1** – Mean diagnostic delay (months) difference between each centers, using t-test student. A p-value < 0.05 was considered statistically significant.

NA = Not applicable

| p-value | Antalya  19.1 ± 26.1 | Hannover  15.4 ± 16.7 | Jena  15.6 ± 21.3 | Lisbon  16.7 ± 21.0 | Warsaw  21.0 ± 31.3 |
| --- | --- | --- | --- | --- | --- |
| Antalya  19.1 ± 26.1 | NA | 0.101 | 0.244 | 0.206 | 0.293 |
| Hannover  15.4 ± 16.7 | 0.101 | NA | 0.946 | 0.432 | **0.021** |
| Jena  15.6 ± 21.3 | 0.244 | 0.946 | NA | 0.612 | 0.100 |
| Lisbon  16.7 ± 21.0 | 0.206 | 0.432 | 0.612 | NA | **0.015** |
| Warsaw  21.0 ± 31.3 | 0.293 | **0.021** | 0.100 | **0.015** | NA |

**Table 2** – Mean time gap (months) between symptoms onset and first medical evaluation, difference between each centers, using t-test student. A p-value < 0.05 was considered statistically significant. NA = Not applicable

| p-value | Antalya  8.1 ± 15.8 | Hannover  5.9 ± 12.6 | Jena  6.3 ± 9.7 | Lisbon  5.2 ± 7.9 | Warsaw  4.7 ± 6.7 |
| --- | --- | --- | --- | --- | --- |
| Antalya  8.1 ± 15.8 | NA | 0.166 | 0.288 | **0.002** | **0.003** |
| Hannover  5.9 ± 12.6 | 0.166 | NA | 0.929 | 0.318 | 0.175 |
| Jena  6.3 ± 9.7 | 0.288 | 0.929 | NA | 0.321 | 0.133 |
| Lisbon  5.2 ± 7.9 | **0.002** | 0.318 | 0.321 | NA | 0.417 |
| Warsaw  4.7 ± 6.7 | **0.003** | 0.175 | 0.133 | 0.417 | NA |

**Table 3** – Mean time gap (months), between first medical evaluation and diagnosis, difference between each centers, using t-test student. A p-value < 0.05 was considered statistically significant. NA = Not applicable

| p-value | Antalya  11.3 ± 20.9 | Hannover  9.7 ± 10.0 | Jena  8.9 ± 17.8 | Lisbon  11.0 ± 19.1 | Warsaw  16.3 ± 29.5 |
| --- | --- | --- | --- | --- | --- |
| Antalya  11.3 ± 20.9 | NA | 0.326 | 0.348 | 0.862 | 0.053 |
| Hannover  9.7 ± 10.0 | 0.326 | NA | 0.644 | 0.345 | **0.002** |
| Jena  8.9 ± 17.8 | 0.348 | 0.644 | NA | 0.321 | **0.023** |
| Lisbon  11.0 ± 19.1 | 0.862 | 0.345 | 0.321 | NA | **0.002** |
| Warsaw  16.3 ± 29.5 | 0.053 | **0.002** | **0.023** | **0.002** | NA |
